# Discordant Vitamin B12 Results in an IgA Nephropathy Patient

**DOI:** 10.1002/jcla.70143

**Published:** 2026-02-06

**Authors:** Oytun Portakal, Nazende Işlak, Muhammed Ş. Binici, Yahya Büyükaşik

**Affiliations:** ^1^ Department of Medical Biochemistry, Faculty of Medicine Hacettepe University Ankara Turkey; ^2^ Department of Hematology, Faculty of Medicine Hacettepe University Ankara Turkey

**Keywords:** immunoassay, interference, macromolecule, polyethylene glycol, vitamin B12 (VitB12)

## Abstract

**Background:**

Discordant Vitamin B12 results can result from immunoassay interferences, potentially leading to unnecessary diagnostic procedures and misdiagnoses if not properly recognized.

**Case Presentation:**

A 76‐year‐old male with a history of IgA nephropathy, hypertension, and other comorbidities presented with unexpectedly elevated total Vitamin B12 levels despite no supplement use and normal hematological and biochemical parameters. Polyethylene glycol (PEG) precipitation demonstrated markedly reduced recovery, indicating the presence of macro‐VitB12. Pretreatment with blocking reagents and exclusion of heterophilic antibodies confirmed the interference. The discrepancy observed between analytical platforms further supported this finding.

**Conclusion:**

Vitamin B12 results should always be interpreted in conjunction with clinical findings. Polyethylene glycol (PEG) precipitation is a cost‐effective and accessible method for detecting macro‐VitB12, particularly in cases where analytical results are inconsistent with the clinical presentation.

**Abbreviations**
CLEIA: chemiluminescent enzyme immunoassay
CMIA: chemiluminescent microparticle immunoassay
FNAB: fine needle aspiration biopsy
HBR: heterophilic blocking reagent
HBT: heterophilic blocking tube
HT: hypertension
IF: intrinsic factor
IgAN: IgA nephropathy
mAB: monoclonal antibody
MMA: methylmalonic acid
PEG: polyethylene glycol
RF: rheumatoid factor
tHcy: total homocysteine
US: ultrasonography
VitB12: vitamin B12

## 1 Case Presentation

A 76‐year‐old male, a retired sculptor, presented to the Geriatrics Department for evaluation of hypertension (HT). His medical history included IgA nephropathy (IgAN), chronic hepatitis B carriage, prostate cancer in remission after radiotherapy, nodular goitre, and motor neuron disease.

IgAN had been diagnosed 20 years earlier following a kidney biopsy performed due to hematuria and proteinuria, after which the patient was managed conservatively. The pathology report showed a mild increase in mesangial cells and matrix, along with focal tubular atrophy and interstitial fibrosis. Immunofluorescence microscopy findings were consistent with IgAN.

Hypertension had been diagnosed 15 years earlier; however, the patient discontinued antihypertensive therapy approximately 2 years ago due to transient visual darkening. His adherence to medical follow‐up was inconsistent. He had previously undergone inguinal hernia repair, nephrolithiasis surgery, and colon polypectomy.

In 2019, a fine‐needle aspiration biopsy (FNAB) was performed because ultrasonography revealed thyroid nodules and elevated thyroid hormone levels, although the patient denied symptoms such as shivering or sweating. The cytological findings were benign. He was not taking any vitamin supplements, and his most recent thyroid function tests were within the reference interval.

Physical examination was unremarkable, with a blood pressure of 145/82 mmHg. Routine biochemical testing showed no proteinuria or hematuria on urine dipstick, and 24‐h urinary protein excretion was within normal limits. Glomerular filtration rate, liver enzymes, and renal function tests were all within reference ranges. Complete blood count values were also normal, though the peripheral smear showed anisocytosis, macrocytosis, and large platelets. Serum total Vitamin B12 (VitB12) exceeded 1107 pmol/L (reference interval: 133–675 pmol/L).

The patient was referred to the Hematology Department to investigate possible hematopoietic malignancy. No underlying disorder was identified, and he was subsequently referred to our laboratory for evaluation of the spurious VitB12 elevation. Key laboratory results from this admission are summarized in Table 1.

**TABLE 1:** Some important results of the patient.

Parameter	Results	Reference range
RBC (10^12^/L)	4.64	4.44–5.61
Hemoglobin (g/dL)	14.5	13.5–16.9
Hematocrit (%)	43.3	40–49.4
MCV (fL)	**93.3**	**81.8–95.5**
RDW (%)	12.6	12–13.6
Ferritin (μg/L)	87.7	20–336
Folate (nmol/L)	38.91	7.05–45.23
Iron (μmol/L)	19.7	8.95–26.86
UIBC (μmol/L)	38.32	22.38–61.78
sTfr (%)	34	20–50
Homocysteine (μmol/L)	**17.7**	**< 15**
MMA (μmol/L)	0.2	< 0.4
Anti parietal antibody	Negative	Negative
Rheumatoid factor (IU/mL)	< 9.25	< 15
ANA	**Positive**	**Negative**
INR	0.95	0.8–1.2
Protein, total (g/L)	69.8	60–85
Albumin (g/L)	46.1	35–50
Creatinine (μmol/L)	74.26	59.23–103.43
24 h urine albumin (mg/day)	28.75	< 30

*Note:* Significant results were bolded.

In the presence of any suspicion of assay interference, a series of laboratory investigations is recommended, including dilution tests, method comparison, immunoglobulin precipitation, blocking of heterophilic antibodies, and size‐exclusion chromatography; however, the latter is not available in all laboratories [1].

Serum VitB12 measurement in our laboratory is performed on the DXI800 analyzer (Beckman Coulter Inc., USA) using a chemiluminescent enzyme immunoassay (CLEIA) based on a competitive immunometric principle employing an intrinsic factor–alkaline phosphatase conjugate, goat anti‐mouse monoclonal IgG, and mouse monoclonal anti–intrinsic factor antibodies bound to paramagnetic particles.

Total immunoglobulin levels (IgG, IgA, IgM) are measured on the BN II system (Siemens Healthcare Diagnostics, UK) by nephelometry using rabbit‐derived antibodies bound to polystyrene beads.

**FIGURE 1:**
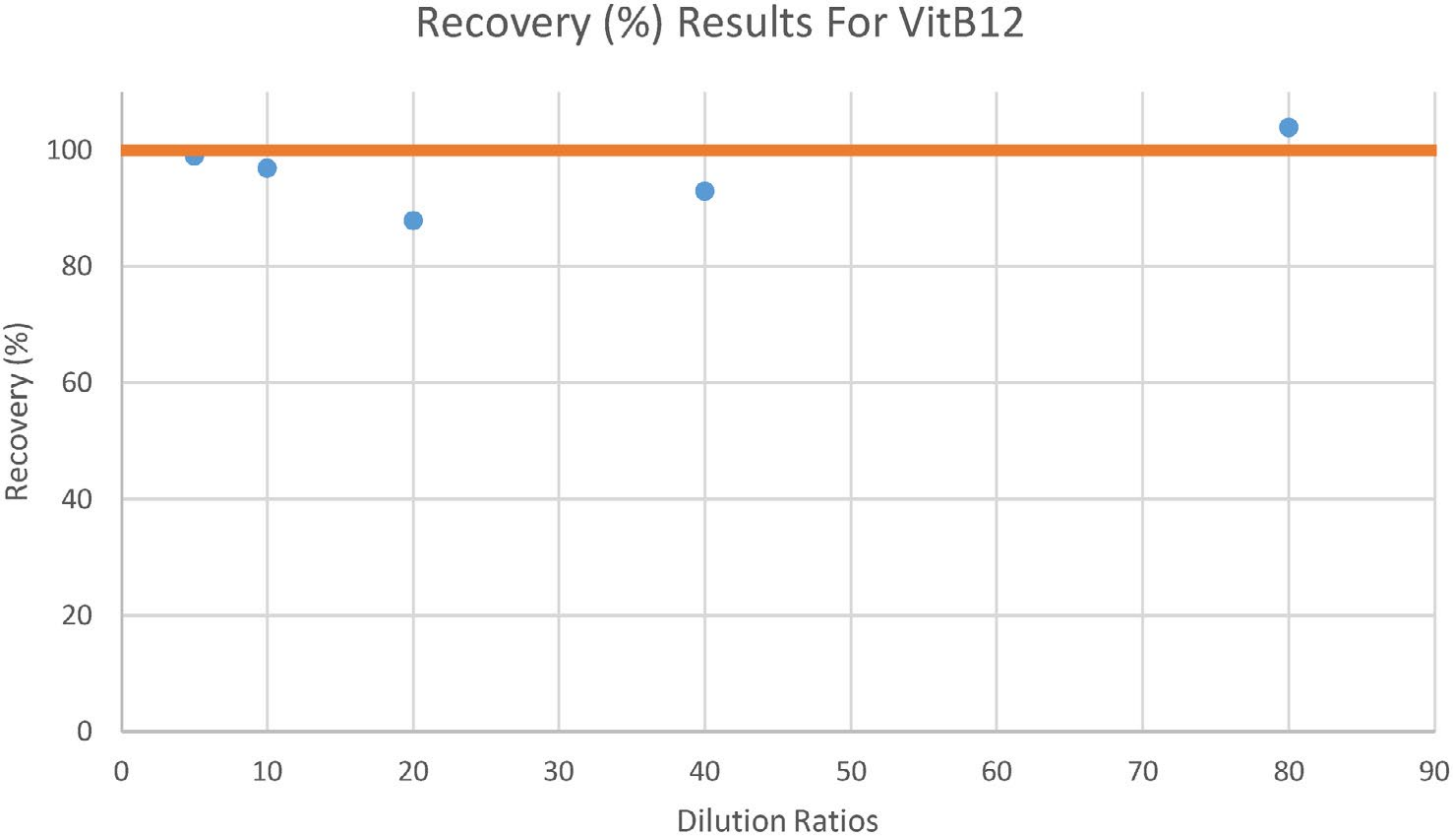
Recovery percentages (%) of total Vitamin B12 across serial dilutions (1:5 to 1:80). Values remain within acceptable linearity limits (88%–104%).

During our laboratory evaluation, a linearity study for VitB12 was initially performed using serial dilutions ranging from 1:5 to 1:80. The ratio of treated to untreated samples was calculated and expressed as a percentage recovery (Rec%). The Rec% values ranged from 88% to 104% (Figure 1). Preservation of linearity can occasionally be observed in some types of assay interference.

For method comparison, serum VitB12 levels were also measured on the Architect i2000SR (Abbott Diagnostics, Ireland) using the CMIA method. Quantification of the CMIA result was not possible due to limited sample volume. A discrepancy was observed between the two methods, suggesting the presence of sample interference (Table 2).

**TABLE 2:** Interference Evaluation Studies for total Vitamin B12 (a, b, c matching parameters analyzed in corresponding autoanalyzers).

	Total VitB12 (pmol/L)^a^	Total VitB12 (pmol/L)^b^	IgG (μmol/L)^c^	IgA (μmol/L)^c^	IgM (μmol/L)^c^
Routine	**4143**	**> 4427**	66.04	15.63	< 0.17
HBT (Rec%)	4241 **(102.37)**	—	78.05 **(118.18)**	17.19 **(110.00)**	< 0.17 (not calculated)
PEG (1:2)^d^ (Rec%)	109.2 **(4.75)**	—	< 9.54 **(< 14.44)**	< 3.13 **(< 40.00)**	< 0.17 (not calculated)
PEG (1:5)^d^ (Rec%)	166 **(7.22)**	—	—	—	—
PEG (1:10)^d^ (Rec%)	324.64 **(14.13)**	—	—	—	—

*Note:* Significant results were bolded.

Abbreviations: HBT, heterophilic blocking tube; PEG, precipitation results after PEG 6000 application.

^a^ Beckman Coulter DXI800 (CLEIA).

^b^ Architect i2000SR (CMIA).

^c^ Siemens BN2 (Nephelometry).

^d^ Dilution ratios after PEG precipitation.

In this patient, serum rheumatoid factor (RF) was within the reference interval, excluding RF‐related antibody interference (Table 1).

Serum immunoglobulins and macromolecular complexes were precipitated using polyethylene glycol (PEG 6000) at a 1:1 dilution (250 g/L in 0.05 mol/L phosphate buffer, pH 7.4) with serum. VitB12 and immunoglobulins were subsequently measured in the supernatant. For VitB12, serial dilutions (1:2–1:10) were applied to minimize viscosity‐related measurement errors. The Rec% values for immunoglobulins confirmed successful precipitation, while VitB12 recovery ranged from 4.75% to 14.13%, suggesting the presence of macromolecular complexes in the sample (Table 2) [2].

In addition, pretreatment with a blocking agent was performed using heterophilic blocking tubes (HBT; Scantibodies, USA). After a 1 h incubation at room temperature in the blocking tubes, serum VitB12 and immunoglobulin levels were analyzed. No significant change in recovery (Rec%) was observed (Table 2).

## 2 Discussion

VitB12 (Cbl) is an essential molecule involved in fatty acid and amino acid metabolism, cell proliferation, nucleotide synthesis, and DNA methylation. However, mammals lack the ability to synthesize this compound and must obtain it through dietary sources. VitB12 deficiency, most commonly resulting from malabsorption or insufficient dietary intake, leads to pernicious anemia accompanied by neurological manifestations such as ataxia and paresthesia, as well as hematological abnormalities including megaloblastic anemia and pancytopenia. Conversely, elevated VitB12 levels have been associated with various neoplasms, liver diseases, acute promyelocytic leukemia, polycythemia vera, chronic eosinophilic leukemia, and vitamin supplementation [3, 4]. Therefore, it is crucial to confirm that an elevation is genuine before proceeding with the differential diagnosis.

The most commonly used technique for determining VitB12 levels is the immunoassay, with a cut‐off value for marginal deficiency of < 221 pmol/L [4]. In addition, methylmalonic acid (MMA) and total homocysteine (tHcy) are important biomarkers of VitB12 status, useful for detecting subclinical deficiency [4].

Immunometric assays are susceptible to interference because they rely on a delicate equilibrium for optimal antibody–analyte binding. Interferences may be either exogenous or endogenous. Exogenous factors include issues related to reagent or calibrator quality, device malfunctions such as pipetting errors, temperature instability, or inadequate washing. These can generally be prevented through appropriate quality control and correct analyzer operation. Endogenous interferences, however, may arise from poor sample quality, antibodies directed against the analyte or reagent antibody, macromolecular complexes, medications, or the hook effect [1]. The impact on results depends on both the source of interference and the analytical method used. For VitB12 measurement, most clinical laboratories employ a competitive assay; in the presence of heterophilic antibodies, this can lead to falsely elevated results [5].

Polyethylene glycol (PEG 6000) is a linear polymer of ethylene glycol with strong water‐absorbing properties. Immunocomplexes have reduced solubility in aqueous solutions due to their larger molecular size. PEG exploits this property by decreasing the availability of free water, thereby precipitating such complexes [6]. However, this method is not specific for targeted complexes; it can also precipitate monomeric analytes, reagent proteins, and alter antibody–analyte interactions. In addition, precipitation of IgA is only partial [1]. For these reasons, optimal cut‐off values for PEG precipitation should be established for each analyte and analytical platform. For VitB12, unlike prolactin, no consensus exists regarding the optimal cut‐off value to identify macrocomplex formation. Only a small number of clinical cases have been reported, and further studies are warranted [3].

Gel filtration chromatography is considered the gold standard for evaluating the presence of macromolecular complexes. However, it is not readily available in most clinical laboratories because of its high cost and technical complexity. Consequently, PEG precipitation remains a practical and cost‐effective alternative for assessing most analytes [2, 7].

Heterophilic antibodies are human antibodies directed against animal antibodies from which reagent antibodies are derived, and they are predominantly of the IgG class. Such antibodies may develop in individuals living in rural areas, working in animal farming, or having frequent animal exposure. They are most often formed against murine antibodies (human anti‐mouse antibodies, HAMA) but can also target antibodies from other species such as bovine, equine, rat, or goat (human anti‐animal antibodies, HAAA). Heterophilic antibodies may interfere with assays by various mechanisms, such as binding to reagent antibodies and preventing analyte interaction, or binding to immobilized antibodies and hindering complex dissociation during wash steps. Heterophilic blocking reagents (HBR) or heterophilic blocking tubes (HBT) are formulated to neutralize such antibodies. The patient had no history of animal farming or pet ownership, and HBT testing was negative; thus, the presence of heterophilic antibodies was excluded [8].

Rheumatoid factors (RF) are IgM‐type antibodies that can bind assay antibodies and are commonly found in patients with autoimmune diseases such as rheumatoid arthritis; they may also increase in infectious conditions. These interferences produce assay effects like those caused by heterophilic antibodies. Therefore, evaluation for autoimmune diseases or infections and measurement of RF are recommended [1]. In this patient, RF levels were within the reference range.

Monoclonal antibodies (mAbs) are produced by cloned cell lines and are widely used in the diagnosis and treatment of various cancers and autoimmune or autoinflammatory disorders. These therapeutic antibodies may cross‐react with reagent antibodies and interfere with immunoassays; hence, their use should be considered during clinical evaluation [1]. This patient had no history of monoclonal antibody therapy.

Intrinsic factor (IF) autoantibodies can be detected in some patients with pernicious anemia. These antibodies prevent VitB12 from binding to IF, resulting in increased circulating concentrations. Reports indicate that Beckman Coulter systems are not affected by this type of interference [9].

We could not determine the precise source of the macromolecule—whether it originated from immune complexes or from anti–transcobalamin II antibodies, the latter being rare. However, identifying the exact source would not influence clinical decision‐making for this patient.

This patient had well‐controlled IgA nephropathy, normal renal function, and no evidence of hypertension, hematuria, proteinuria, neoplasia, or liver disease. He was not taking any vitamin supplements; therefore, assay interference was suspected. PEG precipitation confirmed the presence of macro‐VitB12, with markedly reduced recovery.

Based on the available literature and our findings, normal or even elevated VitB12 levels do not exclude B12 deficiency when clinical suspicion exists [10]. Clinical correlation of laboratory results with peripheral smear findings and patient symptoms remains essential in the evaluation of anemia. When results are discordant, clinicians should consider potential assay interference and consult the laboratory to prevent misdiagnosis and unnecessary investigations.

## 3 Points to Remember

1. Please consider the presence of macro‐VitB12 in patients with high serum VitB12 levels, which is inconsistent with the clinical course.
2. In such cases, request laboratory consultation and contact the laboratory physician.
3. By PEG precipitation method, the presence of macro‐VitB12 will be quickly demonstrated in the lab. However, there is no consensus on the cut‐off value for macro‐VitB12 detection.

## 4 Questions to Consider

1. How is total VitB12 measured?
2. What are the causes of Megaloblastic Anemia?
3. What do you expect to see in a blood smear in megaloblastic anemia?
4. What is the course of action when laboratory results do not match clinical findings?
5. Which studies should be performed when evaluating analytical interference in the laboratory?

## Consent

Written informed consent for publication of the patient's clinical details and accompanying images was obtained. The signed consent form will be provided to the Editor upon request.

## Conflicts of Interest

The authors declare no conflicts of interest.

## Data Availability

Data sharing is not applicable to this article as no new datasets were generated or analyzed during the current study. Patient information is provided in a de‐identified manner within the manuscript.
